# Editorial: Digital health and real-world evidence in onco-hematological patients

**DOI:** 10.3389/fonc.2023.1330407

**Published:** 2023-11-14

**Authors:** Claudia Vener, Laura Lopez-Perez, Maria Fernanda Cabrera-Umpiérrez, Giuseppe Fico

**Affiliations:** ^1^ Epidemiology and Prevention Unit, Department of Epidemiology and Data Science, Fondazione Istituto di Ricovero e Cura a Carattere Scientifico (IRCCS) Istituto Nazionale dei Tumori, Milan, Italy; ^2^ Life Supporting Technologies Research Group, Department of Photonics and Bioengineering, Telecomunication Engineering School, Universidad Politécnica de Madrid, Madrid, Spain

**Keywords:** clinical practice, digital health, hematological malignancies, real-world evidence, representativeness-generalizations

Digital health, as defined by the World Health Organization (WHO), encompasses electronic health (e-health) and, in general, the use of advanced computer sciences, including big data and artificial intelligence (AI). In this context, digital health enhances our ability to study and treat onco-hematological patients in real-world (RW) settings by collecting and sharing RW data from electronic records. The ultimate goal is to support clinical and translational research. Furthermore, digital health RW studies can compile data from numerous cases, providing added value when analyzing rare hematological malignancies.

Digital health solutions address several unmet needs. These include providing easy access to care, reassuring patients, increasing adherence to clinical protocols, enhancing treatment efficacy, reducing hospitalizations, and optimizing healthcare resources. Additionally, AI can support the remote monitoring of cancer patients, assisting healthcare providers with timely interventions. Notably, current challenges in the field of digital health studies involve issues such as data sharing, interoperability, the promotion of open community data standards, and adherence to international guidelines.

Our Research Topic underscores the significance of RW digital health efforts in studying and managing onco-hematological disorders. For instance, Morabito et al. used deep learning and explainable AI to perform gene selection, predicting the need and timing of therapy in chronic lymphocytic leukemia (CLL). Mangone et al. analyzed the incidence, mortality, and survival rates of hematological malignancies in northern Italian patients with data updated through 2020. Mu et al. proposed new machine learning (ML) models by integrating next-generation sequencing and tumor cell size, improving subtype classification of mature B-cell neoplasms (MBN). Wang et al. developed and validated the platelet-to-albumin ratio as a clinical predictor for diffuse large B-cell lymphomas (DLBCL). Bartolini et al. validated the use of Italian administrative healthcare data to describe the RW utilization of infusive antineoplastic drugs. Finally, Zhuang et al. conducted a RW, multi-centered study in China, revealing that clinicians prefer to prescribe lenalidomide or bortezomib as maintenance therapy in non-transplant newly diagnosed multiple myeloma (MM) patients with high-risk cytogenetic abnormalities, as they are superior to thalidomide.

Despite the immense potential of digital RW data for monitoring and evaluating healthcare patterns, including diagnosis, therapy, and daily clinical patient assistance, certain methodological issues continue to be the subject of debate and are discussed here.

First of all, in the six collected articles, data access and sharing primarily occur upon request. Nowadays, we generate vast volumes of data to fuel data-driven scientific discovery. Unfortunately, not all data can be openly shared due to privacy concerns, ownership issues, technical challenges, and trust considerations, hindering research progress and data utility. In medical research, data sharing plays a crucial role in promoting discovery, innovation, transparency, reproducibility, and trust in scientific findings. The COVID-19 pandemic has further accelerated the demand for data sharing, with increasing calls for the rapid dissemination, evaluation, integration, and analysis of new medical research results (1).

Secondly, some digital health projects fail to address clinical needs relevant to clinicians, patients, or public health decision makers. In many cases, they appear to be technical efforts focused on developing statistical or mathematical techniques without effectively solving clinical problems or addressing essential questions. In contrast, papers included in this Research Topic address specific needs. For example, Morabito et al. contributed to the management and treatment of CLL patients. Mangone et al. provided essential epidemiological information on onco-hematological disorders in northern Italy. Mu et al. enhanced the classification of MBN. Wang et al. proposed a clinical predictor for DLBCL. Bartolini et al. validated Italian administrative healthcare data, which is valuable for the public health system. Zhuang et al. contributed to the management of MM patients in a RW Chinese setting.

Thirdly, it’s important to note that while statistical analysis methods like Cox models remain widely used, ML techniques are gradually gaining traction. Some studies in this Research Topic (Mu et al.; Morabito et al.) have utilized ML methods such as Random Forest, K-Nearest Neighbors, Naive Bayes, and Neural Networks. Nevertheless, the widespread adoption of these advanced techniques in this field appears to be in its early stages.

Fourthly, validation is a crucial step in the development of both statistical and ML models as it ensures their ability to generalize to unseen data. Internal validation methods, such as cross-validation, provide an initial assessment of model performance, while external validation, which tests the model on entirely new data, offers a more robust measure of its generalizability. Despite their importance, these validation techniques were not extensively utilized in the studies within this Research Topic. Mu et al. stands out for performing both internal and external validation for the ML techniques, and Wang et al. applied them in their developed Cox models. This may be attributed to a lack of awareness or insufficient data.

After our comprehensive evaluation of the papers’ quality, we considered the issue of translating results into clinical practice. Within our Research Topic, we have identified certain studies that highlight the potential for this translation (Mangone et al.; Mu et al.), while other studies have begun the process of validating their procedures in RW healthcare systems (Bartolini et al.). However, some studies are still in the process of validation (Morabito et al.; Wang et al.; Zhuang et al.).

While progress has been made in applying ML techniques and addressing relevant clinical needs, there is still room for improvement in several areas. These include model validation and adherence to best practice guidelines, such as TRIPOD(-AI) and PROBAST(-AI) (2) for data-driven model reporting, as well as SPIRIT(-AI) and CONSORT(-AI) (3) for designing protocols for clinical studies. These improvements are essential to better address the gaps identified in this Research Topic.

Furthermore, a significant effort should be made to emphasize unmet clinical needs, taking a global perspective and, above all, incorporating aspects related to the patient’s quality of life.

## Author contributions

CV: Conceptualization, Methodology, Supervision, Writing – original draft, Writing – review & editing. LL-P: Conceptualization, Methodology, Supervision, Writing – original draft, Writing – review & editing. MC-U: Supervision, Writing – review & editing. GF: Conceptualization, Supervision, Writing – review & editing.
